# A Review of* Oenanthe javanica* (Blume) DC. as Traditional Medicinal Plant and Its Therapeutic Potential

**DOI:** 10.1155/2019/6495819

**Published:** 2019-04-01

**Authors:** Chuan-li Lu, Xiu-fen Li

**Affiliations:** ^1^Bioengineering Laboratory, Guangdong Bioengineering Institute (Guangzhou Sugarcane Industry Research Institute), Guangdong Academy of Sciences, Guangzhou 510316, China; ^2^Guangdong Key Lab of Sugarcane Improvement & Bio-Refinery, Guangzhou 510316, China; ^3^Yunnan Agricultural University, Kunming 650000, China

## Abstract

*Oenanthe javanica*, popularly known as water dropwort, has long been used in various ethnomedical systems in Asia, especially in China, Korean, and Japan, for treating various chronic and acute hepatitis, jaundice, alcohol hangovers, abdominal pain, and inflammatory conditions. The present review aims to provide a general report of the available literature on traditional uses, phytochemical, pharmacological, nutritional, and toxicological data related to the* O. javanica* as a potential source of new compounds with biological activities. Considering phytochemical studies, coumarins, flavonoids and flavonoid glycosides, organic acids, and polyphenols were the main classes of compounds identified in the whole plant which were correlated with their biological activities such as hepatoprotective, anti-inflammatory, immune enhancement, ethanol elimination, antioxidant, antiviral, neuroprotective, anti-cancer, anticoagulant, anti-fatigue, hypoglycemic, cardiovascular protection, analgesic, and insecticidal activities.

## 1. Introduction

Before modern drugs began to take shape in the medical care industry, people were highly dependent on conventional or traditional medicine, which have been recognized by the World Health Organization as reliable medicinal sources for therapeutic activities [1, 2]. Medicinal plants, the “backbone” of traditional medicine, are utilized today by more than 3.3 billion people in the less developed countries [3].

*Oenanthe javanica* (Blume) DC. (Apiaceae), which is a small perennial herb, has been cultivated in tropical and temperate regions of Asia for thousands of years and has long been used as a folk remedy for alleviating a wide spectrum of diseases. A variety of biological activities of* O*.* javanica* have been reported, including hepatoprotective [4, 5], anti-inflammatory [6, 7], immune enhancement [8], ethanol elimination [9], antioxidant [10], and antiviral [11]. Phytochemical assessments have revealed that* O*.* javanica* contains coumarins [12], flavonoids and flavonoid glycosides [13], and polyphenols [4]. In addition, toxicity studies have demonstrated that* O*.* javanica* does not exhibit acute or genetic toxicity [14, 15]. However, oral administration of dry* O*.* javanica* power could significantly increase the rate of mice sperm deformity and even induce reduction of weight and food consumption at a high dose [15]. Moreover, total phenolics acid extract from* O*.* javanica* at a high dose (equivalent to 20 times of recommended human clinical dose) showed a reversible subchronic toxicology [16].

Thus, this review is aimed at elucidating the biological and pharmacological activities, as well as toxicology of* O*.* javanica*.

## 2. Plant Description

Since* O*.* javanica* distributes and is cultivated in many areas of Asia and Australia, each group of local people have a specific name for this plant as listed in Table 1 [10, 17–26]. In China, this plant is commonly known as* Shui qin*,* Shui qin cai*,* Shui ying*, and so on [17, 18], while it is known as* Seri* in Japan [19–21]. The plant is called* Minari* [22],* Selom *[23–26], and* Phak-chilom* [10] in Korea, Malaysia, and Thailand, respectively.

This plant is a small perennial herb and grows up to 10-80 cm with fibrous roots that emerge from all nodes. The stems are light green, terete, glabrous, vertically veined, and hollow, which are more or less erect, but sometimes sprawl. Its basal petioles are 5-10 cm long. The leaves are aromatic and glabrous and have a sheath covering the stem. The blade is oblong-ovate with 1-2 pinnate, while ultimate segments are 5–50 mm long with 5–20 mm broad either ovate or rhombic-ovate shape, margins serrate. Cauline leaves gradually reduced upwards, smaller, becoming sessile on expanded sheaths. There are 5 white petals and 5 stamens for its flowers, with umbels 3–5 cm across, peduncles 2–16 cm, bracts absent or occasionally 1 bract, and rays 6–16(–30), 1–3 cm, subequal or unequal; bracteoles 2–8, linear, 2–4 cm, as long as pedicels; umbellules ca. 20-flowered; pedicels 1.5–4 mm. Calyx teeth ca. 0.5 mm. Its fruit is subglobose or ovoid, ca. 2.5 × 2 mm, while dorsal and intermediate ribs are slightly corky-thickened [27, 28].

## 3. Ethnomedicinal Uses of* O. javanica*

Generally,* O. javanica* is a valuable herbal plants consumed and used by East Asian countries for both food and various medicinal purposes (Table 2) [4, 5, 11, 13, 17, 23, 29–41]. For example, the flower and stem (or the aerial parts) of this plant are commonly used in China for the treatment of various types of chronic and acute hepatitis [11, 17, 29, 30]. It is also used in China for jaundice [13, 23, 35, 36], fever [4, 35, 37], hypertension [13, 32, 35–37], abdominal pain, and urinary difficulties [4, 35, 36, 39, 40], as well as for eliminating pathogenic wind [17, 29]. Similarly, treatments for fever and hypertension are also common medicinal uses in Korea and Malaysia [23, 32–34]. In Korea, this plant is also used for treating alcohol hangovers and inflammatory conditions [11, 39, 41].

Besides medicinal applications,* O*.* javanica* has also been widely consumed as a dietary product. The whole plant or the aerial parts of* O*.* javanica* in Malaysia are a well-known vegetable and freshly consumed as the main ingredient in local famous food “ulam,” which constitutes an important part of the food intake among the local peoples especially the Malay and Indigenous communities [23–26], while, in Korea, this plant is widely used in salad and soups [22]. In Japan,* O. javanica* named “seri” is one of the ingredients of the symbolic dish, Nanakusa-no-sekku, consumed in the Japanese spring-time festival [19–21].

## 4. Phytochemistry of* O. javanica*/Chemical Components

### 4.1. Flavonoids and Flavonoid Glycosides

**Figure 1:**
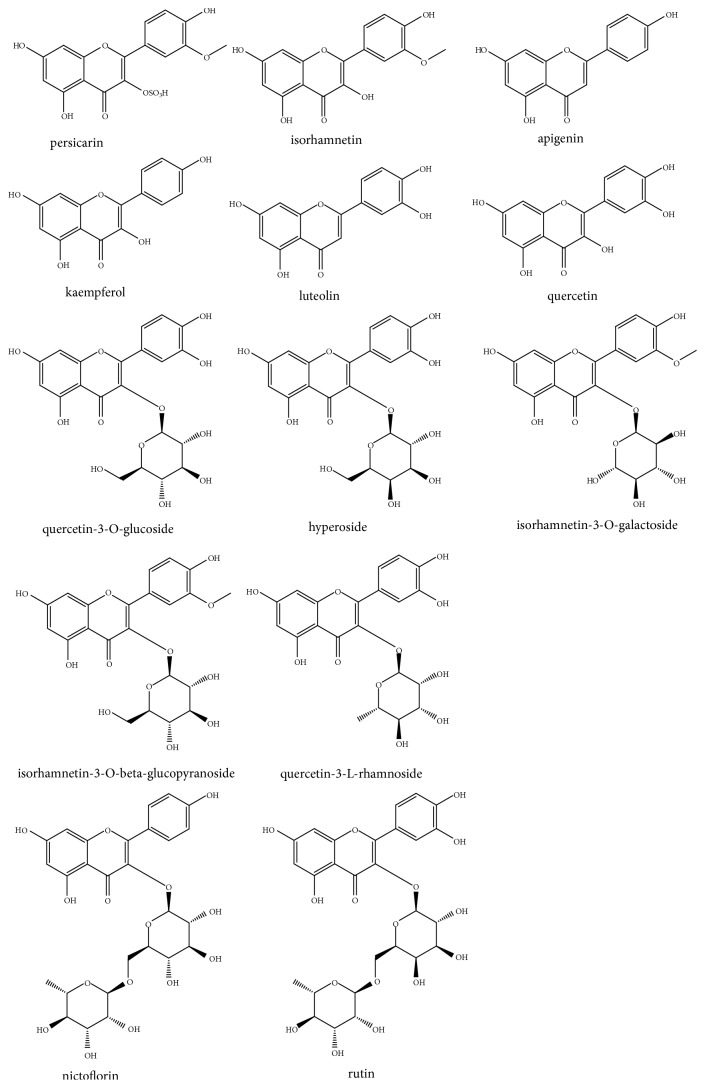
Structures of flavonoids and flavonoid glycosides isolated from* O. javanica*.

Flavonoids and flavonoid glycosides are abundant in* O. javanica*, and more than ten flavonols have been isolated and identified from* O. javanica* thus far, including apigenin [3], isorhamnetin-3-*O*-*β*-*D*-glucopyranoside [18], quercetin [37], isorhamnetin-3-*O*-galactoside [39], afzelin [41], persicarin, isorhamnetin, hyperoside [56], luteolin [57], kaempferol, rutin, nictoflorin, and quercetin-3-L-rhamnoside [58, 59] (their structures are depicted in Figure 1). In general, all of the flavonoids and flavonoid glycosides obtained from this plant have free phenolic hydroxyl groups in the 5, 7, and 4′-position. Most of them are substituted in the 3 and 3′-position. For all flavonoid glycosides obtained from this plant, aglycons are attached at 3-position.

### 4.2. Coumarins

**Figure 2:**
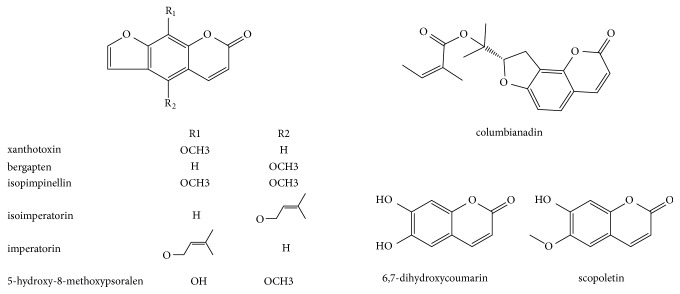
Structures of coumarins isolated from* O. javanica*.

Approximately nine coumarins have been identified from* O. javanica*, namely, xanthotoxin, bergapten, isopimpinellin [12], sioimperatorin, imperatorin, columbianadin, 5-hydroxy-8-methoxypsoralen, 6,7-dihydroxycoumarin, and scopoletin [18] (the structures are presented in Figure 2). Most of these components are the linear furanocoumarins, with the 5- and 8-positions being substituted by methoxyl and isoamylenoxyl groups.

### 4.3. Phenolic Constituents

**Figure 3:**
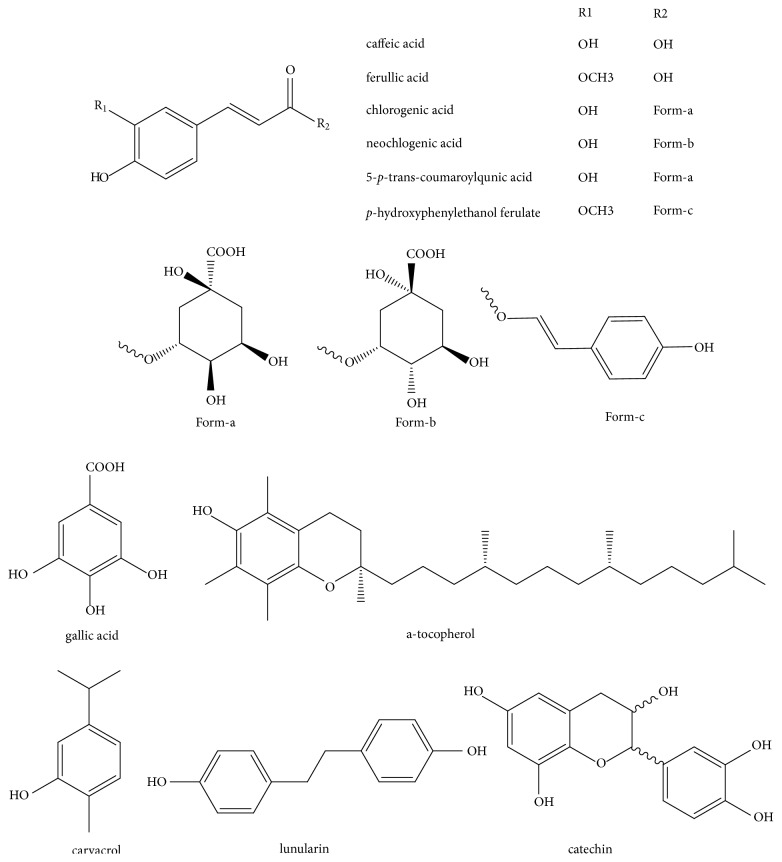
Structures of phenolic constituents isolated from* O. javanica*.

Phenolics are also abundant in* O. javanica*, including neochlorogenic acid [4], chlorogenic acid [4, 5, 11, 60], caffeic acid [4, 5, 48, 57, 60], gallic acid [4, 57], *α*-tocopherol [10, 61], lunularin,* p*-hydroxyphenylethanol ferulate, 5-*p*-trans-coumaroylquinic acid [18], carvacrol, ferullic acid [57], and catechin [45] (the structures are presented in Figure 3). For phenolic acids and ester identified from* O*.* javanica*, most of them are caffeic acid derivatives. The content of total phenolic acids in* O. javanica* from Korean was 88.9 ± 0.46 mg GAE/g [45] and in* O. javanica* from Guizhou Province of China was 131.5-173.2 mg/g [62]. In addition,* O. javanica* extract contains *α*-tocopherol (146.8 mg/kg) [61], gallic acid (0.9 ± 0.23 mg/g), catechin (1.2 ± 0.19 mg/g), chlorogenic acid (227.1 ± 0.62 mg/g), and caffeic acid (4.0 ± 0.35 mg/g) [45].

### 4.4. Volatile Oils

The volatile constituents are extracted with steam distillation, vacuum simultaneous steam distillation and solvent extraction, and solid-phase microextraction, and 59 chemical constituents have been identified with gas chromatography-mass spectrometer and computer retrieval technique, including 28 hydrocarbons, 16 alcohols, 8 aldehydes, 4 esters, 2 ethers, and 1 ketone. Furthermore, by using the gas chromatography-olfactometry,* p*-cymene has been identified as a character-impact aroma-active compound of* O*.* javanica*, and *α*-terpinolene, *α*-terpinene, (*E*)-caryophyllene, and (*Z*,*E*)-*α*-farnesene also play significant roles in the aroma of* O*.* javanica *[42]. Lee et al. identified 15 compounds representing 100% of volatile oil, mainly including *β*-caryophyllene, *δ*-cadinene, *β*-bisabolene, *α*-terpinolene, *γ*-terpinene, and *α*-amorphene [44]. Zhang et al. reported 16 volatile constituents consisting of 96.46% of oil, and the principal constituents were eudesma-4(14),11-diene,2,3-dihydro-3-methyl-3-benzofuran-methanol, limonene, and allylphenoxyacetate [43]. The identified volatile constituents from* O*.* javanica *are presented in Table 3.

### 4.5. Other Ingredients

**Figure 4:**
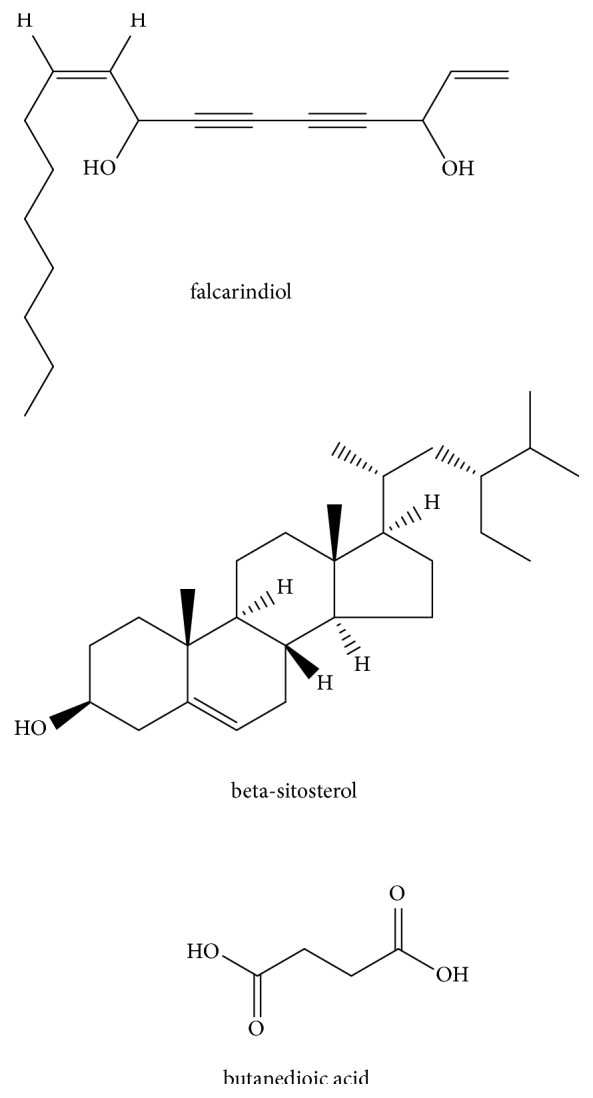
Structures of other ingredients isolated from* O. javanica*.

Other compounds, not list above, have been isolated from* O*.* javanica* including butanedioic acid [4], *β*-sitosterol [18], and falcarindiol [21]. Their structures are shown in Figure 4.

## 5. Nutrient Constituent of* O. javanica*

The fresh* O. javanica* plant has wide variety nutrients such as carbohydrates, proteins, vitamins, and fat, as well as mineral micronutrients, as shown in Table 4 [10, 45–47].* O*.* javanica *has high iron content, followed by kalium, calcium, natrium, and magnesium, which are useful for patients with mineral deficiencies problems. The plant has moisture content of up to 88% [10] and a total ashes value of 8.9% [45] suitable for body hydration.

## 6. Pharmacological Activities

### 6.1. Hepatoprotective Effect

Although* O*.* javanica* has been traditionally used as a Dai ethnic medicine for various liver diseases, the scientific evidence that justifies its usage has only recently been reported (summarized in Table 5). The significantly hepatoprotective effect of* O*.* javanica* extracts has been demonstrated on both cell lines and animal models.

Treatment with boiling water extract of* O. javanica *(at dose equivalent to 12 g fresh material/kg) showed a significantly suppression effect on the elevation of serum bilirubin level and the degeneration and necrosis of hepatic cells in *α*-naphthylisocyanate-stimulated Wistar rats, but no effect on serum alanine aminotransferase (ALT) level [49]. In addition, the hepatoprotective effects of total phenolics from* O. javanica* have been reported on different liver injury models, including _*D*_-galactosamine- and carbon tetrachloride (CCl_4_)-induced acute liver injury in mice [4, 50, 51], CCl_4_-induced chronic hepatic fibrosis in rats [51], a-naphthylisothiocyanate-induced liver jaundice in rats [52], and high-sugar high fat-induced non-alcoholic fatty liver in rats [53].

Moreover, fermented* O. javanica* extracts, in which caffeic acid and chlorogenic acid were the major constituents, have been reported to dose-dependently inhibit tert-butylhydroperoxide-induced HepG2 cells death and lactate dehydrogenase leakage, as well as prevent the increase of hepatic enzyme markers ALT, aspartate aminotransferase (AST), and gene expressions of cytochrome P450 enzyme (CYP)2E1, CYP4A2 and PPAR*γ* in CCl_4_-induced hepatic damage in rats [5], while other studies demonstrated that pretreatment by ethanol extract of* O. javanica* (500 *μ*g/ml) or its active component (caffeic acid, 250 mM) could significantly reduce hydrogen peroxide (H_2_O_2_)-induced cellular toxicity in human liver hepatocellular carcinoma HepG2 cell line [48] and counteract the oxidative stresses through increasing the expressions of the endogenous enzymatic antioxidants such as superoxide dismutase (SOD), catalase (CAT), and glutathione peroxidase (GPx) in the liver cells of rat [32].

A recent study indicated that persicarin isolated from* O. javanica* could effectively prevent diabetes-induced liver damage by attenuating oxidative stress and inflammation response under hyperglycemic conditions [41]. Another study in a different experimental model of liver disease, nonalcoholic fatty liver disease (NAFLD), revealed that both boiling water extract and* n*-butanol extracts of* O. javanica* pretreatment effectively lowered plasma triglyceride and glucose levels [54]. In addition, the underlying mechanism of hepatoprotective effects of* O. javanica* was demonstrated to be attributed to the improvement of antioxidant capacity and the inhibition of hepatic stellate cell activation and hepatic malondialdehyde production, and consequent attenuation of inflammatory responses and chemical-induced liver injury [55].

The hepatoprotective activity of* O*.* javanica* extract was also caused by selective inhibition activity of cytochrome P_450_ and consequently affected various xenobiotic metabolism. Investigation in HepG2 also revealed that the root extract of* O. javanica* could significantly elevate the mRNA expressions (by 68% and 102%, respectively) and protein levels (by 112 and 157%, respectively) of CYP1A1 and CYP1A2 and these effects were much more pronounced than those of leaf and stem extracts [12]. Of note, this study provides additional evidence that the levels of major coumarin derivatives determined by GC–MS, including xanthotoxin, bergapten, and isopimpinellin, were significantly higher in root extract than in leaf or stem extracts, which might be responsible for those effects, suggesting dietary exposure to* O. javanica* may modulate phase I enzymes and thereby affect various xenobiotic metabolism [12]. Specifically, hyperoside (quercetin-3-*O*-galactoside), a flavonoid isolated from* O. javanica*, was reported to selectively inhibit the cytochrome P_450_ isoform and strongly decreased CYP2D6 activity at dose-, but not time-dependent manner in human liver microsomes (HLMs). In this case, hyperoside strongly inhibits CYP2D6-catalyzed dextromethorphan* O*-demethylation, with IC_50_ values of 1.2 and 0.81 *μ*M after 0 and 15 min of preincubation and a* Ki* value of 2.01 *μ*M in HLMs, respectively. Moreover, hyperoside decreased CYP2D6-catalyzed dextromethorphan* O*-demethylation activity of human recombinant cDNA-expressed CYP2D6, with an IC_50_ value of 3.87 *μ*M using a cocktail probe assay. However, no inhibition of other CYPs by hyperoside was observed. These results suggest that hyperoside isolated from* O. javanica* might cause herb-drug interactions when coadministered with CYP2D6 substrates [22].

### 6.2. Anti-Inflammatory Effect

It has been reported that* O*.* javanica* extracts possessed significant anti-inflammatory activities, while isorhamnetin, hyperoside, and persicarin were revealed as its main active components for anti-inflammatory effect.

The extract of* O*.* javanica* showed a significant inhibitory effect on nitric oxide production (IC_50_ < 61 *μ*g/ml) in interferon gamma/lipopolysaccharide stimulated RAW264.7 cells assay, without cytotoxicity [23], as well as an attenuate effect in phorbol 12-myristate 13-acetate-treated THP-1 or bone marrow derived macrophages cells on secretion of interleukin-1*β* and formation of Asc pyroptosome resulting from NOD-like receptor (NLR)P3, NLRC4 and absent in melanoma 2 (AIM2) inflammasome activation without interruption of cytokine transcription [6]. In addition, its main component, isorhamnetin, a 3′-*O*-methylated flavonoid, exhibited a selectively inhibitory effect on NLRP3 and AIM2 inflammasome activation and expression of proinflammatory cytokine, while hyperoside, another component of* O*.* javanica*, selectively interrupted NLRC4 and AIM2 inflammasome activation but did not alter cytokine expression. In addition, both of them showed an obvious suppression effect on caspase-1 secretion [6].

Furthermore, the anti-inflammatory effect of isorhamnetin in lipopolysaccharide-activated RAW264.7 cells was confirmed to be partly mediated by inhibiting the mitogen activated protein kinase (MAPK)-nuclear factor-kappa B (NF-*κ*B) signaling pathway [7]. The* in vivo* anti-inflammatory activity of isorhamnetin was also confirmed in carrageenan-induced rats, which showed that oral administration of isorhamnetin (10 or 30 mg/kg) could markedly inhibit carrageenan-induced paw swelling, inflammatory cell infiltration, and proinflammatory gene expression in rats [7]. Moreover, the anti-inflammatory activities of isorhamnetin-3-*O*-galactoside and persicarin isolated from the aerial parts of* O*.* javanica* were demonstrated on high mobility group box 1 (HMGB1)-mediated inflammatory response, and the results showed that both of them could inhibit the releasing of HMGB1 and HMBG1-dependent inflammatory responses in human endothelial cells, as well as HMBG1-mediated hyperpermeability and leukocyte migration in mice [63, 64].

### 6.3. Enhancing Immunity

The effects of* O*.* javanica* extract on immune function/regulation were evaluated in normal and hydrocortisone-induced immunodepressed mice [8, 65].

Total flavone from* O*.* javanica* has been demonstrated to possess an upregulatory effect on cell immunity, humoral immunity, and nonspecific immunity in hydrocortisone-induced immunodepressed mice model, as it could obviously increase the carbon clearance index, the serum hemolysin content, and spleen and thymus index and enhance delayed-type hypersensitivity [8]. In line with these findings, the enhancement effect of total phenolics acid extract of* O*.* javanica* on immune function was also demonstrated on normal mice, by markedly increasing the content of serum hemolysin and interleukin-2, promoting proliferative response of splenic T-lymphocyte induced by concanavalin A, and improving the clearance rate of charcoal particles in peripheral blood in mice [65].

### 6.4. Ethanol Elimination/Alcohol Detoxication

Alcohol abuse, especially excess alcohol consumption or alcohol hangovers, is related to impaired liver function. According to surveys, prolonged use is related to increase the risk of liver disease, such as cirrhosis and liver failure.

A hot-water extract of* O*.* javanica* injection exhibited a rapidly reducing effect on the plasma ethanol level in ethanol-treated New Zealand white rabbit. In addition, oral administration of* O*.* javanica* extract and its* n*-butanol fraction could eliminate up to 44% and 70% of the plasma ethanol, respectively (compared to orally ethanol-treated mice). Specifically, the* n*-butanol fraction exhibited the strongest activity in eliminating plasma alcohol. These data indicated that* O. javanica* extract is effective in overcoming alcohol intoxication by accelerating ethanol metabolism [9]. Moreover, the methanol extract of* O. javanica* and persicarin isolated from the aerial parts of the plant possessed a dose-dependent stimulatory effect on alcohol-metabolizing enzymes, including alcohol dehydrogenase, aldehyde dehydrogenase, and the microsomal ethanol-oxidizing system in ethanol-treated rats [56].

### 6.5. Antioxidant Activity

The antioxidant activity of* O. javanica* have been evaluated using several types of assays, such as scavenging 2,2-diphenylpicrylhydrazyl (DPPH) radical, oxygen radical absorbance capacity (ORAC), ferric reducing antioxidant power (FRAP), and Xanthine oxidase assays. The antioxidant activity of* O. javanica* methanol extract was firstly revealed by Huda-Faujan et al. using FRAP tests [25]. In addition, the 95% ethanol extract of the dried leaves exhibited a radical scavenging for DPPH and inhibitory effect on SOD activity, with the inhibition rates of 56.87 ± 1.43% and 73.51 ± 0.54% at concentration of 10 mg/ml, respectively. However, there was no significant correlation between antioxidant activities and its phenolic contents [66]. Similarly, the antioxidant properties of methanol extract from* O. javanica* performed by using DPPH assay showed that IC_50_ value was 87.42 ± 0.64 *μ*g/ml [23]. Recently, Kongkachuichai et al. showed that hydrophilic ORAC and FRAP activities of* O. javanica* were about 9000 and 2000 *μ*mol Trolox equivalent /100 g fresh weight [10]. To date, studies dealing with the antioxidant activity of* O. javanica* related to phenolics are not conclusive.

### 6.6. Antiviral Effect

The anti-hepatitis B virus (HBV) effects of* O. javanica* were conducted in human hepatoma (HepG2.2.15 cells) culture system and HBV-infected duck models. Total phenolics, flavones, and ethyl acetate extracts from* O. javanica* have been revealed to possess significant anti-HBV activities. Flavones extract from* O. javanica* showed a significant inhibitory effect on HBsAg and HBeAg secretion in HepG2.2.15 cells within nontoxic concentrations, and on duck hepatitis B virus (DHBV)-DNA levels in HBV-infected duck model with concentrations of 0.50 and 1.00 g/kg. Results indicated that the half value of toxic concentration (TC_50_) and maximum nontoxic concentration (TC_0_) was 2.28 g/L and 1.00 g/L, respectively. The maximum inhibition peak of viremia was at dose of 1.00 g/kg and reached 54.3% on day 5 and 64.5% on day10, respectively [13].

Total phenolic acid from* O. javanica* (OJTP) also showed a strong inhibition effect on HBV-DNA (inhibition rate: 62.3%, 47.7%) and _CCC_DNA (inhibition rate: 62.7%, 61.3%) expressions at 250 and 500 mg/L at day 8, respectively, in HepG2.2.15 cells. Besides, this inhibition rate remained high after 3 days of* O. javanica* treatment [67]. In addition, OJTP exhibited dose-dependent suppression activities against the production of the HBeAg and HBsAg in HepG2.2.15 cells line [11, 67], and DHBV-DNA replication in ducks [11]. The maximum inhibition peak of viremia was at dose of 0.20 g/kg and reached 64.10% on day 5 and 66.48% on day 10, respectively. Histopathological evaluation of the liver revealed significant improvement by OJTP. No matter whether 5-day or 10-day administration, or 3 days after 10-day administration, the groups treated with OJTP (500, 250, 125 mg/kg/d) had significantly inhibitory action on DHBV-DNA induced hepatitis model in Peking ducks. Histopathological evidences from the results showed that OJTP treated hepatic lobules were regular, and the denaturation, dropsy, and necrosis of cells were trivial. Meanwhile the hepatic cells are confused and disorderly, oxyphilous denaturation, and dropsy and necrosis is obviously surrounding hepatic lobules. These data indicated that OJTP has significantly inhibitory effects on DHBV-DNA and can protect duck livers from damage in virus hepatitis [68]. Similar results were reported by Huang et al. [69, 70]. In DHBV infected duck primary hepatocytes culture, water extract of* O. javanica* was shown to potentially inhibit DHBV-DNA levels with the inhibition rate of 64% at 2500 *μ*g/ml and half value of effective concentration (EC_50_) was 1120.8 *μ*g/ml, which was much less than its TC_50_ (10000 *μ*g/ml), indicating* O. javanica* is a hopeful drug for controlling DHBV [69]. The mean inhibition rate of* O. javanica* on DHBV-NDA polymerase was 75.5% (at dose of 10000 *μ*g/ml)* in vitro* and 73.3% (at dose of 8 g/kg) i*n vivo*, indicating strong inhibition effect of* O. javanica* on DHBV-NDA polymerase. In addition, IC_50_ (407 *μ*g/ml) was far less than TC_50_ (>10000 *μ*g/ml) on liver cell [70].

The inhibition effect of ethyl acetate extract of* O. javanica* on HBsAg and HBeAg and its toxicity was demonstrated in the HBV transfected HepG2.2.15 cells. The results showed that the TC_50_ of the extract was 2284.73 ± 127.35 *μ*g/ml, and the TC_0_ was 1000 *μ*g/ml. After 6 and 9 days' treatment, the extract significantly inhibited the secretion of HBsAg and HBeAg in HepG2.2.15 cell line at doses of 1000 and 500 *μ*g/ml [71].

### 6.7. Neuroprotective Activity

The neuroprotective activities of* O. javanica* extracts have been uncovered by several studies, which showed that the extracts could improve cell proliferation and neuroblast differentiation, protect neurons from ischemic damage, and maintain antioxidants immunoreactivities [34, 38, 40, 72]. The ethanol extract of* O. javanica* showed an ameliorating effect on cell proliferation and neuroblast differentiation by increasing brain-derived neurotrophic factor immunoreactivity in the dentate gyrus of adolescent rat [40]. Park et al. revealed that treatment with* O. javanica* extract (at a dose of 200 mg/kg) exhibited a protective effect on the hippocampal cornus ammonis 1 pyramidal neurons against cresyl violet induced ischemic damage, and this protective effect is closely associated with increasing or maintaining intracellular antioxidant enzymes such as glutathione peroxidase [34]. The F-box-protein 7 (*FBXO7*) mutations were found in typical and young onset Parkinson's disease, which plays an important role in the development of dopaminergic neurons. Increased stability and overexpression of* FBXO7* may be beneficial to Parkinson's disease. Chen et al. demonstrated that 95% ethanol extract of* O*.* javanica* could, through enhancing* FBXO7* and decreasing tumor necrosis factor receptor-associated factor 2 expression, improve cell viability of both 1-methyl-4-phenylpyridinium ion (MPP^+^)-treated human embryonic kidney-293 (HEK-293) and SH-SY5Y cells, increase proteasome activity in MPP^+^-treated HEK-293 cells, and restore mitochondrial membrane potential in MPP^+^-treated SH-SY5Y cells. Thus, ethanol extract of* O*.* javanica *could be developed as a potential treatment of Parkinson's disease [72]. In addition, Ma et al. documented that persicarin, isolated from n-butanol fraction of* O. javanica*, possessed an obvious neuroprotective activity in glutamate-injured rat cortical cells by reducing calcium influx, inhibiting the subsequent overproduction of nitric oxide and intracellular peroxide, restoring the reduced activities of glutathione reductase and glutathione peroxidase [38].

### 6.8. Anti-Cancer Activity

It was demonstrated that the total phenolics acid extract from* O. javanica* possessed an inhibitory effect on HepG2.2.15 cell proliferation, which could reduce cell growth at S phase [73]. In addition, an* in vitro* migration and invasion assay showed that isorhamnetin, a flavonoid isolated from* O. javanica*, possessed an anti-metastatic effect, which may correlate with its inhibition of reactive oxygen species-mediated hypoxia inducible factor-1*α* (HIF-1*α*) accumulation [74]. It could significantly inhibit cobalt chloride (CoCl_2_)- or hypoxia-induced HIF-1*α* accumulation in human cancer cells (HCT116 and HT29 cell lines), as well as suppress CoCl_2_-induced activity of hypoxia response element reporter gene and HIF-1*α*-dependent transcription of gene such as glucose transporter 1, lactate dehydrogenase A, carbonic anhydrase-IX, and pyruvate dehydrogenase kinase 1. And the inhibitory effect of isorhamnetin on H_2_O_2_-induced HIF-1*α* accumulation was also observed in HEK293 cells.

### 6.9. Anticoagulant/Antithrombotic Activities

Persicarin, isorhamnetin, hyperoside, and isorhamnetin-3-*O*-galactoside, which were isolated from* O. javanica*, were demonstrated to possess significantly antithrombotic activities [39, 75]. All of them could significantly prolong activated partial thromboplastin time and prothrombin time and inhibit both the activities and generations of thrombin and factor X in human umbilical vein endothelial cells (HUVECs). In accordance with these anticoagulant activities, these four compounds also exhibited inhibitory effect on tumor necrosis factor-alpha-induced plasminogen activator inhibitor type 1 (PAI-1) production. Moreover, persicarin and isorhamnetin showed a prolonged effect on bleeding time* in vivo*, while treatment with isorhamnetin-3-*O*-galactoside or persicarin resulted in a significant reduction effect on the ratio of PAI-1/tissue-type plasminogen activator. The anticoagulant and profibrinolytic effects showed that persicarin > isorhamnetin, hyperoside > isorhamnetin-3-*O*-galactoside, which suggest that the sulfonate group of persicarin or the methoxy group of isorhamnetin-3-*O*-galactoside positively regulates its anticoagulatory function.

### 6.10. Anti-Fatigue Effect

The anti-fatigue effect of* O*.* javanica* was studied in a wheel apparatus-induced fatigue mice model, and results indicated that oral administration of extract from* O*.* javanica* (125, 250 and 500 mg/kg per day) for 4 weeks could significantly prolong the exhausted running time, reduce serum levels of lactic acid, malondialdehyde, and urea nitrogen, increase the activities of serum lactate dehydrogenase and superoxide dismutase, and elevate glycogen reserves and hemoglobin concentration in whole blood [76]. And a further study demonstrated that treatment with extract from* O*.* javanica* for 10 days could obviously upregulate the decreases in locomotor activity, function of the axis of hypothalamic-pituitary-adrenal and gonadal axis, and situation of peripheral fatigue and downregulate the elevated levels of central neurotransmitters and radical toward normal values in forcing swimming-induced chronic fatigue syndrome mice model [77]. In addition, Su et al. further demonstrated that treatment with extract from* O*.* javanica* significantly improved hydrocortisone-induced decrease in locomotor activity, cyclic adenosine monophosphate (cAMP), ratio of cAMP/cGMP (cyclic guanosine monophosphate), total testosterone level, and increased of malondialdehyde (MDA) and SOD activity [78].

### 6.11. Hypoglycemic Effect

Oral administration of water extract of* O*.* javanica* (10 and 20 g/kg per day) for 2 days significantly lowered the blood glucose levels in normal mice and alloxan-induced hyperglycemic mice, but did not affect mice hyperglycemia induced by adrenaline [79]. In addition, a further study showed that pretreatment with water extract of* O*.* javanica* (50, 100, and 200 g/kg) could significantly suppress the blood glucose level and improve the decreased content of insulin in streptozotocin (STZ)-induced mice, and these effects may be due to its alleviation of the condition of the degeneration and necrosis of islet cells induced by STZ [80]. Furthermore, 95% ethanol extract of* O*.* javanica* (400 and 800 mg/kg) was also revealed to possess a moderate hypoglycemic activity in STZ-induced diabetic mice model, which could decrease the blood glucose level from 27.6 mM to 20.8 mM and 17.7 mM, respectively [62].

### 6.12. Cardiovascular Protection

The antiarrhythmic effect of* O*.* javanica* was demonstrated in aconitine-induced rats, which indicated that intravenous administration of* O*.* javanica* injection (1.5 g/kg) could significantly increase the threshold levels of ventricular premature, ventricular tachycardia, ventricular fibrillation, and cardiac arrest (respectively, 27%, 22%, 32%, and 19% higher than aconitine along-treated rats) and also make rats arrhythmia induced by barium chloride conversed to sinusrhythm within 6.29 min and keep sinusrhythm for another more 12.73 min, as well as decrease the rate of calcium chloride-induced rat ventricular fibrillation and death by 25% and 50%, respectively [81].

Moreover, persicarin and isorhamnetin-3-*O*-galactoside isolated from* O*.* javanica* were revealed to possess potential therapeutic for treatment of severe vascular inflammatory diseases, which could both, through reducing phorbol 12-myristate 13-acetate-stimulated phosphorylation of p38 MAPK, extracellular regulated kinases 1/2, and* c*-jun* N*-terminal kinase, suppress the expression of tumor necrosis factor-*α* and then inhibit the phorbol-12-myristate-13-acetate, cecal ligation, or puncture-induced endothelial protein C receptor [36].

### 6.13. Antinociceptive/Analgesic Effect

The antinociceptive active of* O. javanica* methanol extract (at 200 mg/kg) was tested in the acetic acid-induced abdominal writhing response in mice, which indicated that* O*.* javanica* extract could reduce about 30% of abdominal constriction, while positive control-aspirin could inhibit about 62% of writing inhibition. Furthermore, the underlying mechanism of antinociceptive effect of* O*.* javanica* extract was demonstrated to be mediated by the suppression of nitric oxide production and reducing the sensitization of the peritoneal nociceptor in mice [23].

### 6.14. Insecticidal Effect

Huo et al. pointed out that* O*.* javanica* extract possesses a marked effect to kill the* tetranychina harti* (Ewing), which supported its usage as a new plant candidate for acaricide [82].

## 7. Toxicological Studies and Adverse Reaction

*O*.* javanica* was documented to be a nontoxic level species by acute toxicity tests, because the maximum tolerated dose of it was higher than 15 g/kg for mice [14, 15, 83]. In an acute toxicity study, a single oral administration of fresh* O*.* javanica* (15 g/kg) did not cause young mice mortality or inductive changes in the ALT, blood glucose, total protein, albumin, urea, and creatinine levels, and no signs of abnormal behavioral changes or toxicity on organs including liver and kidney were observed after 14 days of treatment [14]. Furthermore, in Ames assay (8-5000 *μ*g/vessel) and mouse bone marrow cell micronucleus test (2.50-10.00 g/kg),* O*.* javanica* showed no obvious genetic toxicity [15]. But an increasing effect on the rate of sperm deformity was observed in mice by oral administration of dry* O*.* javanica* power for 5 days (2.50, 5.00, and 10.00 g/kg, 1 g dry power equivalent to 13 g fresh material). Moreover, a subacute toxicity, including weight loss and reduction in food consumption, was also observed in mice by oral administration of dry* O*.* javanica* power for 30 days (5.00 g/kg/day) [15]. For subchronic toxicity assay, total phenolics acid extract from* O*.* javanica* at doses of 1500 and 750 mg/kg (26 weeks) showed no significant effect on rats body weight, food intake, behaviors, blood routine examination (counts of red blood cells, white blood cells, and platelets; percentages of neutrophils, lymphocytes, monocytes, and hemoglobin; and prothrombin time), serum biomarkers (AST, ALT, ALP, total bilirubin, urea nitrogen, Crea, total protein, albumin, total cholesterol, and blood glucose), or organs [heart, liver, spleen, lung, kidney, adrenal, testis (for male rat), ovary (for female rat), and brain]. However, at the dose of 3000 mg/kg, it showed a decreasing effect on weight gain and lymphocyte number, and an increasing effect on neutrophil, but no effect on other tested items. Furthermore, within a 4-week recovery period, the induced toxicity was basically recovered [16].

In addition, a rare case of irritant contact dermatitis owing to* O*.* javanica* was reported by Xia and Li [84], in which a 23-year-old male patient had edematous erythema and bullae appeared on his shoulder, back, and knees where fresh crush of* O*.* javanica* was applied. There was also burning-like epidermal exfoliation on the lesions. The patient felt strong burning pain but no itching and was cured by 7-day anti-inflammatory treatment.

## 8. Conclusions

The present review collectively discussed the ethnomedicinal uses of* O*.* javanica* and the available scientific reports on its phytochemistry, pharmacological activities, and toxicology. It is worth mentioning that although scientific studies of bioactivities of* O*.* javanica* might justify some of its ethnomedicinal claims, the data are insufficient and, to some extent, preliminary. In the future, further systemic studies in humans are necessary. Furthermore, a subchronic toxicology of* O*.* javanica* at high dose (equivalent to 20 times the recommended human clinical dose) was observed in rats, but the potential toxic component and its possible mechanism have not been revealed. It would also be beneficial for* in vivo* and clinical studies to evaluate the toxicity effects on the target organ.

## Figures and Tables

**Table 1:** Common names used for *O*.* javanica* in different countries.

Country	Local name	References
China	Shui qin(水芹)	[17, 18]
Japan	Seri, *セリ*	[19–21]
Korea	Minari, 미나리	[22]
Malaysia	Selom	[23–26]
Thailand	Phak-chilom	[10]

**Table 2:** Ethnomedicinal uses of *O*. *javanica* in different countries.

Traditional uses	Plant parts used	Country	Refs.
Various types of chronic and acute hepatitis	Flowers and stem (or the aerial parts)	China	[11, 17, 29, 30]
Jaundice	Leaves and stems (or the aerial parts)	Korean	[5, 31–34]
Jaundice	NI or Whole plant	China	[13, 23, 35, 36]
Eliminating pathogenic wind	NI	China	[17, 29]
Fever	NI	China	[4, 35, 37]
Fever, hypertension, epidemic influenza, haematuria	Whole plant	Malaysia	[23]
Fever	NI	Korean	[32–34]
Hypertension	NI	China	[13, 32, 35–37]
Hypertension, abdominal pain	NI	Korean	[31, 33, 34, 36, 38]
polydipsia diseases	NI	China	[13, 17, 29, 37]
polydipsia diseases	NI	Korean	[5]
abdominal pain, leucorrhea	NI	China	[4, 35, 36, 39]
overcoming alcohol hangovers	NI	Korea	[39]
Leucorrhea, mumps, urinary difficulties or infections	NI	Korean	[33, 34]
Mumps, urinary difficulties or infections	NI	China	[4, 35, 36, 39, 40]
inflammatory conditions	NI	Korean	[11, 41]

NI: not indicated. The plant parts are written as in the original literature source.

**Table 3:** Volatile compounds identified from *O*.* javanica*.

Compounds	Values (%)	Refs.
1-(4-methylphenyl)-ethanone	-	[42]
1,2,3,4,4,5,6,8-octahydro-4,8-dimethyl-2-(1-methylethenyl)- [2R-(2,4,8)]-naphtha-ene	0.89	[43]
1-ethenyl-1-methyl-2,4-bis(1-methylethenyl)- [1S-(1*α*,2*β*,4*β*)]-cyclohexane	0.10	[43]
1-octen-3-yl	0.1	[42]
2,3-dihydro-3-methyl-3-benzofuranmethanol	2.94	[43]
2,6,6,9-tetramethyl-tricyclo-[5.4.0.02.8]-undec-9-ene	0.23	[43]
2,6-nonadienal	-	[42]
2-heptanol	-	[42]
3,7,11-trimethyl-(*E*)-1,6,10-dodecatrien-3-ol	0.66	[43]
3,7-dimethyl-1,3,6-octa-triene	0.62	[43]
3-octanyl	0.1	[42]
4,11,11-trimethyl-8-methylene-[1R-(1R,4Z,9S)]- bicyclo-[7.2.0] undec-4-ene	0.81	[43]
6-butyl-1,4-cycloheptadiene	0.46	[43]
Allylphenoxyacetate	80.17	[43]
Bicycloelemene	-	[42]
Borneol	0.2	[42]
Bornyl	0.1	[42]
Butylatedhydroxytoluene	0.28	[43]
Camphene	-	[42]
Carvacry methyl	0.5	[42]
Caryophyllene oxide	1.49	[44]
(*E*)-1-hexenol	-	[42]
(*E*)-2-hexenal	-	[42]
(*E*)-2-hexenol	-	[42]
(*E*)-2-nonenal	-	[42]
(*E*)-caryophyllene	6.1	[42]
	20.46 (*β*-caryophyllene)	[44]
	0.12 (*α*-caryophyllene)	[43]
(*E*)-farnesol	2.4	[42]
(*E*)-*β*-farnesene	2.3	[42]
(*E*)-*β*-ocimene	0.7	[42]
(*E*,*E*)-2,4-hexadienal	-	[42]
(*E,E*)-*α*-farnesene	3.9	[42]
Epoxyterpine	0.1	[42]
Eudesma-4(14),11-diene	6.83	[43]
Falcarinol	2.1	[42]
Germacrene D	-	[42]
Hexanal	-	[42]
Hinesol	0.14	[43]
Limonene	8.6	[42]
	2.79 (*α*-limonene)	[44]
	1.63	[43]
Limonene oxide	-	[42]
Linalool	-	[42]
	0.64 (*β*-linalool)	[44]
Methanol	10.77	[44]
Methyl phenyl	-	[42]
Neophytadiene	0.9	[42]
	2.83	[44]
Nerolidol	-	[42]
	2.11	[44]
Nonanal	0.1	[42]
Octanol	0.1	[42]
*p*-cymen-8-ol	-	[42]
*p*-cymene	3.8	[42]
	2.91 (cymene)	[44]
Phenylacetaldehyde	-	[42]
Sphathulenol	1.1	[42]
Terpinen-4-ol	0.8	[42]
Thymyl methyl	0.7	[42]
(*Z*)-2-pentenol	-	[42]
(*Z*)-3-hexenal	-	[42]
(*Z*)-3-hexenol	0.1	[42]
(*Z*)-*β*-ocimene	0.2	[42]
(*Z,E*)-*α*-farnesene	5.9	[42]
*α*-amorphene	0.2	[42]
	5.38	[44]
*α*-cadinol	1.5	[42]
*α*-copaene	0.7	[42]
	2.52	[44]
*α*-humulene	1.4	[42]
*α*-phellandrene	0.3	[42]
*α*-pinene	0.7	[42]
*α*-selinene	3.8	[42]
*α*-terpinene	0.1	[42]
*α*-terpineol	0.1	[42]
*α*-terpinolene	2.5	[42]
	7.05	[44]
*β*-elemene	0.6	[42]
*β*-farnesene	6.21	[44]
*β*-myrcene	1.0	[42]
	0.47	[43]
*β*-pinene	3.4	[42]
	1.77	[44]
	0.11	[43]
*β*-selinene	4.2	[42]
*γ*-terpinene	21.7	[42]
	6.85	[44]
*δ*-cadinene	4.7	[42]
	14.46	[44]

-: unstated.

**Table 4:** Nutritional composition of *O*. *javanica*.

Nutritional	Values	Refs.
Calcium (Ca)	133.07 ± 3.07 mg/100 g fresh weight	[10]
	82.6 ± 14.3 mg/100 g 70% EEOJ^*∗*^	[45]
	160 mg/100 g fresh weight	[46]
Copper (Cu)	82.6 ± 0.1 mg/100 g 70% EEOJ	[45]
	0.10 mg/100 g fresh weight	[46]
Ferrum (Fe)	1.35 ± 0.19 mg/100 g fresh weight	[10]
	1.35 mg/100 g fresh weight	[47]
	1.9 ± 4.2 mg/100 g 70% EEOJ	[45]
	6.90 mg/100 g fresh weight	[46]
Kalium (K)	414.52 ± 5.81 mg/100 g fresh weight	[10]
	10372.9 ± 15.3 mg/100 g 70% EEOJ	[45]
	212.00 mg/100 g fresh weight	[46]
Magnesium (Mg)	29.99 ± 1.09 mg/100 g fresh weight	[10]
	198.3 ± 11.2 mg/100 g 70% EEOJ	[45]
	16.00 mg/100 g fresh weight	[46]
Manganese (Mn)	2.6 ± 0.9 mg/100 g 70% EEOJ	[45]
Natrium (Na)	134.6 ± 5.9 mg/100 g 70% EEOJ	[45]
	40.60 mg/100 g fresh weight	[46]
Phosphorus (P)	60.55 ± 5.69 mg/100 g fresh weight	[10]
	865.1 ± 10.3 mg/100 g 70% EEOJ	[45]
	32.00 mg/100 g fresh weight	[46]
Selenium (Se)	0.81 mg/100 g fresh weight	[46]
Zinc (Zn)	2.6 ± 0.7 mg/100 g 70% EEOJ	[45]
	0.38 mg/100 g fresh weight	[46]
Crude Ash	8.9 ± 2.5 g/100 g 70% EEOJ	[45]
Moisture	88.29 ± 0.18 g/100 g fresh weight	[10]
	59.42 g/100 g fresh weight	[47]
	8.8 ± 1.4 g/100 g 70% EEOJ	[45]
Carbohydrate	3.42 ± 0.15 g/100 g fresh weight	[10]
	44.7 ± 2.3 g/100 g 70% EEOJ	[45]
	1.80 g/100 g fresh weight	[46]
Fibre	8.74 g/100 g fresh weight	[47]
	0.90 g/100 g fresh weight	[46]
Total soluble sugar	9.72 g/100 g fresh weight	[47]
Glucose	2.4 ± 1.3 mg/100 g 70% EEOJ	[45]
Fructose	4.3 ± 0.9 mg/100 g 70% EEOJ	[45]
Crude lipid	27.8 ± 0.9 g/100 g 70% EEOJ	[45]
Fat	0.46 ± 0.01 g/100 g fresh weight	[10]
Protein	2.88 ± 0.12 g/100 g fresh weight	[10]
	25.10 g/100 g fresh weight	[47]
	9.8 ± 1.5 g/100 g 70% EEOJ	[45]
	2.60 g/100 g fresh weight	[46]
*β*-carotene	1687.11 ± 62.48 *μ*g/100 g fresh weight	[10]
	0.38 mg/100 g fresh weight	[46]
Lutein	7439.11 ± 287.24 *μ*g/100 g fresh weight	[10]
Total polyphenol	239.23 ± 6.10 mg GAE/100 g fresh weight	[10]
Vitamin A	5.36 mg /100 g fresh weight	[46]
Vitamin C	3.29 ± 0.17 mg AA/100 g fresh weight	[10]
	180 mg /100 g fresh weight	[47]
	21.9 ± 0.5 mg/100 g 70% EEOJ	[45]
	5.00 mg /100 g fresh weight	[46]
Vitamin E	0.83 ± 0.03 mg/100 g fresh weight	[10]
	0.32 mg /100 g fresh weight	
Vitamin B1	1.2 ± 1.1 mg/100 g 70% EEOJ	[45]
	0.36 mg /100 g fresh weight	[46]
Vitamin B2	2.3 ± 0.6 mg/100 g 70% EEOJ	[45]
	0.09 mg /100 g fresh weight	[46]
Vitamin B6	0.04 ± 0.1 mg/100 g 70% EEOJ	[45]
Nicotinic acid	8.3 ± 1.2 mg/100 g 70% EEOJ	[45]

*∗* EEOJ: ethanol extracts of *O*. *javanica*.

**Table 5:** Summary of hepatoprotective activities of different parts, extracts, and active compounds of *O*.* javanica*.

Extract/active compound (Dose)	Model	Effect	Ref.
Total phenolics extract (125, 250, and 500 mg/kg)	_D_-gala-induced mice liver injury	Improving the survival rate of mice, suppressing the elevation of serum enzymatic markers (ALT, AST, ALP, GGT) and total bilirubin.	[4]
Fermented extract of *O. javanica* (1, 10, 100, and 1000 *μ*g/mL, as well as 50, 200, 400 mg/kg)	*t*-BHP induced HepG2 cell cytotoxicity and CCl_4_ induced rat liver damage	Inhibiting cell death and lactate dehydrogenase leakage in* t*-BHP-induced HepG2 cell line; ameliorating the serum levels of ALT and AST, gene expressions of CYP2E1, CYP4A2, and peroxisome proliferator-activated receptor gamma in CCl_4_-induced liver injury rats.	[5]
Root extract of *O. javanica* (100 to 1600 *μ*g/ml)	HepG2 cells	Up-regulating the mRNA expressions and protein levels of CYP1A1 and CYP1A2 in HepG2 cells.	[12]
Isorhamnetin-3-*O*-galactoside and hyperoside (0.5, 1, or 2 *μ*M, and 0.1 to 17.5 *μ*M)	Pooled HLMs and cocktail probe assay	Hyperoside inhibited CYP2D6-catalyzed dextromethorphan *O*-demethylation, with IC_50_ values of 1.2 and 0.81 *μ*M after 0 and 15 min of pre-incubation and a *Ki* value of 2.01 *μ*M in HLMs, respectively. Hyperoside decreased CYP2D6-catalyzed dextromethorphan *O*-demethylation activity of human recombinant cDNA-expressed CYP2D6, with an IC_50_ value of 3.87 *μ*M.	[22]
70% ethanol extract (0.5% of pellet diet weight)	Healthy male SD rats	Increasing the expressions of SOD1 and SOD2, CAT, and GPx in the rat liver cells	[32]
Persicarin (2.5 and 5 mg/kg)	Diabetes-induced mice liver damage	Decreasing the elevated serum and hepatic glucose levels, suppressing the increased oxidative stress parameter (reactive oxygen species, peroxinitrite, and thiobarbituric acid-reactive substance), nicotinamide adenine dinucleotide phosphate oxidase subunit (Nox-4 and P47phox) and inflammatory related makers (NF-*κ*B, AP-1, TGF-*β*, COX-2, and iNOS), as well as inhibiting the serum levels of ALT and AST	[41]
80% ethanol extract and caffeic acid (500 *μ*g/ml and 250 mM)	H_2_O_2_-induced HepG2 cells cytotoxicity	Suppressing H_2_O_2_-induced cellular toxicity in HepG2 cells	[48]
Boiling water extract of *O. javanica* (equivalent to 12 g fresh material/kg)	ANIT-induced hepatitis in Wistar rats	significantly decreasing the elevated serum bilirubin level and alleviated the condition of the degeneration and necrosis of hepatic cells.	[49]
Total phenolic acid (OJTPA, 500, 250, and 125 mg/kg)	_D_-gala-induced liver injury in mice	Inhibiting MDA concentration and raised SOD activity, as well as alleviated the damaged condition of livers in _D_-gala-induced mice.	[50]
Total phenolic acid (OJTPA, 500, 250, and 125 mg/kg)	CCl_4_-induced liver damage in mice	Significantly inhibiting the increasing serum ALT and AST levels.	[51]
Total phenolic acid (OJTPA, 300, 150, and 75 mg/kg)	ANIT-induced jaundice in Wistar rats	Obviously decreasing the elevated levels of total bilirubin, serum MDA and SOD activities.	[52]
Total phenolic acid (OJTPA, 300, 150, and 75 mg/kg)	NAFLD in rats	Obviously ameliorating the hepatocyte fatty degeneration and hepatocyte necrosis.	[53]
Boiling water extract and its n-butanol fraction [8% (v/w)]	NAFLD in male SD rats	Obviously ameliorating hepatic fat accumulation, hyperglycemia, and dyslipidemia.	[54]
Total phenolic acid (OJTPA, 300, 150, and 75 mg/kg)	CCl_4_-induced rats hepatic fibrosis	OJTPA treatment significantly suppressed the CCl_4_-induced hepatic fibrosis in rats by increasing the rats liver antioxidant capacity and inhibiting the of activation of hepatic stellate cell.	[55]

ANIT: *α*-naphthylisocyanate.

_D_-Gala: _D_-galactosamine.

## Abbreviations

AIM2: Absent in melanoma 2
ALP: Alkaline phosphatase
ALT: Alanine aminotransferase
AST: Aspartate aminotransferase
cAMP: Cyclic adenosine monophosphate
cGMP: Cyclic guanosine monophosphate
CAT: Catalase
CCl_4_: Carbon tetrachloride
CoCl_2_: Cobalt chloride
CYPs: Cytochrome P450 enzymes
DHBV: Duck hepatitis B virus
DPPH: 2,2-Diphenylpicrylhydrazyl radical
EC_50_: Half value of effective concentration
EEOJ: Ethanol extracts of* O. javanica*
FBXO7: * F*-box-protein 7
FRAP: Ferric reducing antioxidant power
GPx: Glutathione peroxidase
H_2_O_2_: Hydrogen peroxide
HBeAg: Hepatitis B e antigen
HBsAg: Hepatitis B surface antigen
HBV: Hepatitis B virus
HIF-1*α*: Hypoxia-induced hypoxia inducible factor-1*α*
HLMs: Human liver microsomes
HMGB1: High mobility group box 1
HUVECs: Human umbilical vein endothelial cells
IC_50_: Half maximal inhibitory concentration
MAPK: Mitogen activated protein kinase
NF-*κ*B: Nuclear factor-kappa B
MDA: Malondialdehyde
MPP^+^: 1-Methyl-4-phenylpyridinium ion
HEK-293: Human embryonic kidney-293 cell (ATCC No. CRL-1573)
NALFD: Nonalcoholic fatty liver disease
NLR: NOD-like receptors
OJTP: Total phenolic acid from* O. javanica*
ORAC: Oxygen radical absorbance capacity
PAI-1: Plasminogen activator inhibitor type 1
SOD: Superoxide dismutase
STZ: Streptozotocin
TC_50_: Half value of toxic concentration.
